# Changes in Gut Microbiota and Short‐Chain Fatty Acids in Different Stages of Alzheimer's Disease

**DOI:** 10.1002/alz70855_105512

**Published:** 2025-12-24

**Authors:** Sithara Dissanayaka, Thilini Jayasingh, Hamid R Sohrabi, Stephanie R Rainey‐Smith, Karen Scott, Ralph N Martins, Warnakulasuriya Mary Ann Dipika Binosha Fernando, AIBL Research Group

**Affiliations:** ^1^ Centre of Excellence for Alzheimer's Disease Research and Care, School of Medical and Health Sciences, Edith Cowan University, Joondalup, Western Australia, Australia; ^2^ Alzheimer's Research Australia, Nedlands, Western Australia, Australia; ^3^ The Charles Perkins Centre, The University of Sydney, Camperdown, NSW, Australia; ^4^ Macquarie Medical School, Macquarie University, Macquarie Park, NSW, Australia; ^5^ School of Psychology, Murdoch University, Murdoch, Western Australia, Australia; ^6^ Murdoch University, Murdoch, Western Australia, Australia; ^7^ Alzheimer's Research Australia, Perth, Western Australia, Australia; ^8^ Centre for Healthy Ageing, Murdoch University, Murdoch, Western Australia, Australia; ^9^ Edith Cowan University, School of Medical and Health Sciences, Centre of Excellence for Alzheimer's Disease Research & Care, Joondalup, Western Australia, Australia; ^10^ Alzheimer's Research Australia, Perth, Western Australia, Australia; ^11^ Murdoch University, Perth, Western Australia, Australia; ^12^ Microbial Ecology Group, Rowett Institute, University of Aberdeen, Abrdeen, Abrdeen, United Kingdom; ^13^ Lions Alzheimer's Foundation, Perth, Western Australia, Australia; ^14^ School of Medical and Health Sciences, Edith Cowan University, Perth, Western Australia, Australia; ^15^ Edith Cowan University, Perth, Western Australia, Australia; ^16^ Macquarie University, Sydney, NSW, Australia; ^17^ Centre for Ageing, Cognition and Wellbeing, Macquarie University, North Ryde, NSW, Australia

## Abstract

**Background:**

Gut microbiota and their metabolites, particularly short‐chain fatty acids (SCFAs), play a vital role in the gut‐brain axis, and have been associated with neurodegenerative diseases like Alzheimer's disease (AD). However, the changes in gut microbiota composition and SCFA levels during the progression of AD are not yet well understood. This study seeks to investigate these variations to gain deeper insights into their potential role in disease development.

**Method:**

This study examined changes in gut microbiota and SCFA across three groups; Cognitively unimpaired individuals with low amyloid‐beta ((CU) Aβ Low (*n* =  71)), CU Aβ High (*n* =  19), and those diagnosed with mild cognitive impairment (MCI) or AD (Disease Group (DG), n = 10). Participants were selected from well characterised cohorts and underwent Pittsburg compound B‐positron emission tomography to determine cerebral amyloid status. Faecal microbiota composition was assessed using shotgun metagenomics, while faecal SCFA concentrations were quantified via Gas Chromatography‐Mass Spectrometry (GC‐MS). Associations between taxa and SCFAs were assessed using Spearman correlation and MaAsLin2.

**Result:**

Firmicutes, Proteobacteria, and Bacteroidetes exhibited significant correlations with SCFAs across all groups. In the CU Aβ Low and Disease Group (DG), Firmicutes showed Positive correlations with butyric acid. Group‐specific patterns included negative correlations between Bacteroidetes and propionic acid in the DG group, a positive correlation between Firmicutes and total SCFAs in the CU Aβ Low group, and a positive correlations between Proteobacteria and Actinobacteria with butyric acid in the CU Aβ High group, alongside notable interactions with isovaleric acid. Furthermore, specific taxa such as *Corynebacterium falsenii* (Phylum: Actinobacteria), *Ruthenibacterium lactatiformans* (Phylum: Firmicutes), and *Streptomyces capitiformicae* (Phylum: Actinobacteria) showed significant associations with SCFAs, particularly propionic acid and butyric acid.

**Conclusion:**

These findings suggest that changes in gut bacteria and their metabolites vary at different stages of AD. Key results show that certain bacteria, such as Firmicutes, Bacteroidetes, and Proteobacteria, are linked to SCFAs, especially butyric acid, which plays a role in gut and brain health. This suggests that modifying gut bacteria could help regulate SCFA levels and potentially slow the progression of AD. However, more research is needed to fully understand this connection.

